# An Avirulent Strain of Soybean Mosaic Virus Reverses the Defensive Effect of Abscisic Acid in a Susceptible Soybean Cultivar

**DOI:** 10.3390/v11090879

**Published:** 2019-09-19

**Authors:** Mazen Alazem, Kristin Widyasari, Kook-Hyung Kim

**Affiliations:** 1 Department of Agricultural Biotechnology, College of Agriculture and Life Sciences, Seoul National University, Seoul 08826, Korea; m.alazem@gmail.com (M.A.); kristinwidyasari@gmail.com (K.W.); 2 Plant Genomics and Breeding Institute, College of Agriculture and Life Sciences, Seoul National University, Seoul 08826, Korea; 3 Research Institute of Agriculture and Life Sciences, College of Agriculture and Life Sciences, Seoul National University, Seoul 08826, Korea

**Keywords:** extreme resistance, plant virus, *Rsv3*, soybean mosaic virus, callose, RNA-silencing pathway, abscisic acid, plant–virus interactions

## Abstract

In soybean cultivar L29, the *Rsv3* gene is responsible for extreme resistance (ER) against the soybean mosaic virus avirulent strain G5H, but is ineffective against the virulent strain G7H. Part of this ER is attributed to the rapid increase in abscisic acid (ABA) and callose, and to the rapid induction of several genes in the RNA-silencing pathway. Whether these two defense mechanisms are correlated or separated in the ER is unknown. Here, we found that ABA treatment of L29 plants increased the expression of several antiviral RNA-silencing genes as well as the *PP2C3a* gene, which was previously shown to increase callose accumulation; as a consequence, ABA increased the resistance of L29 plants to G7H. The effect of ABA treatment on these genes was weaker in the rsv3-null cultivar (Somyungkong) than in L29. Besides, G5H-infection of Somyungkong plants subverted the effect of ABA leading to reduced callose accumulation and decreased expression of several RNA-silencing genes, which resulted in increased susceptibility to G5H infection. ABA treatment, however, still induced some resistance to G7H in Somyungkong, but only *AGO7b* was significantly induced. Our data suggest that *Rsv3* modulates the effect of ABA on these two resistance mechanisms, i.e., callose accumulation and the antiviral RNA-silencing pathway, and that in the absence of *Rsv3*, some strains can reverse the effect of ABA and thereby facilitate their replication and spread.

## 1. Introduction

Soybean mosaic virus (SMV), a widespread pathogen of soybeans, is a single-stranded RNA virus that belongs to the Potyviridae family and encodes 11 proteins [1,2]. Several SMV strains with the ability to escape recognition of defense-related proteins in different soybean cultivars have been reported [1,2]. Most of the resistant loci identified in soybean plants are non-Toll interleukin receptor, nucleotide-binding site, leucine-rich repeat (TIR-NBS-LRR) type of *R* genes, or shortly termed (NLR) [1]. To date, researchers have identified four dominant resistance genes (*Rsv1*, *Rsv3*, *Rsv4*, and *Rsv5*) that are effective against several SMV strains [1,2,3,4].

Soybean L29 is an isoline from cv. Williams with an SMV-resistance Rsv3 locus derived from cv. Hardee [5,6]. The Rsv3 locus was previously mapped on chromosome 14 between markers A519F/R and M3Satt, a 154-kbp region that contains a five-member family of coiled-coil (CC) NBS-LRR genes [7]. The Rsv3 locus confers extreme resistance (ER) to several SMV strains including G5H but is ineffective against the virulent strain G7H [1,8]. Rsv3 recognizes a specific region in the cytoplasmic inclusion (CI) in strains G5H and G7, and this recognition triggers ER against these avirulent strains [2,9,10]. The virulent strain G7H, however, escapes this recognition due to slight differences in its CI region, and this SMV strain is, therefore, able to replicate and spread systemically without alerting any of the basal defense arrays [10,11]. An Rsv3 candidate gene, Glyma.14g204700, was recently cloned and characterized as encoding an NLR-type resistance protein; when G5H was inoculated on rsv3-null plants (cv. Lee74) that transiently express Glyma.14g204700, G5H RNA replication was drastically decreased [3]. On the other hand, several soybean cultivars have high-quality traits but lack strong resistance against SMV, and these include the Somyungkong (SMK) cultivar, which is an rsv3-null line that is susceptible to both G5H and G7H [12].

Abscisic acid (ABA) plays a pivotal role in plant–pathogen interactions as well as in modulating plant responses to various stimuli [13,14]. Few antiviral defenses, such as callose accumulation and the RNA-silencing pathway are partially regulated by ABA [15,16,17]. Part of the Rsv3-mediated ER is attributed to the induction of ABA at an early stage of infection, which induces resistance against G5H [8,11]. Genes involved in the ABA pathway and the RNA-silencing pathway, as well as those responsible for inducing callose, were unchanged or even downregulated when soybean cultivar L29 was infected with G7H. However, treating L29 plants with ABA before inoculation with G7H enhanced resistance to this virulent strain [11]. In *Arabidopsis*, the ABA pathway regulates the expression of several Argonaute (AGO), dicer like (DCL), and RNA-dependent RNA polymerase (RDR) genes in the antiviral RNA-silencing pathway [13,15,16,17]. This regulation was prominent for *AGO2* and *AGO3*, because they contribute to defense against bamboo mosaic virus through the ABA-signaling pathway [15,16]. It, therefore, seems that ABA tunes antiviral defenses by controlling callose accumulation as well as the expression of several genes in the antiviral RNA-silencing pathway.

Recent research indicates that immune networks in plants consist of three layers: i) sensor receptors that recognize pathogen effectors; ii) co-receptors that are required by the sensor receptors (helper receptors) and that transduce the effector-recognition event to a downstream signaling network; and iii) a downstream signaling network that generates immune responses [18,19]. NLR receptors are one class of immune receptors, and Rsv3 fits the definition of a sensor receptor. However, helper receptors and the downstream-associated network are still unknown in soybean plants or for the *Rsv3* gene.

As noted earlier, Rsv3-plants express ER against G5H infection, and when they are treated with ABA, they exhibit induced resistance against G7H infection. The defense mechanisms and their amplitude that are triggered in response to SMV infection in *rsv3*-null plants, however, are unknown. In this work, we compared the antiviral defense mechanisms (callose accumulation and the RNA-silencing pathway) in response to virulent and avirulent strains of SMV, in L29 vs. SMK plants (i.e., *Rsv3* vs. *rsv3*-null plants), and with conditions under which these defense responses are usually boosted by ABA treatment in other plants such as *Arabidopsis*. Although ABA treatment of L29 plants enhanced resistance to G7H infection by increasing the expression of PP2C3a (thereby increasing callose accumulation) and by inducing the antiviral RNA-silencing pathway, these effects were subverted in response to G5H in SMK plants, i.e., the combination of ABA treatment and G5H inoculation reduced callose accumulation and the expression of several genes in the RNA-silencing pathway of SMK plants. These findings highlight a critical role for the *Rsv3* resistance gene in modulating ABA-mediated antiviral defenses, and implies that G5H strain has the ability to interfere with the ABA signaling pathway.

## 2. Materials and Methods

### 2.1. Plant Materials and Growth Conditions

*Rsv3*-containing L29 soybean plants and *rsv3*-null SMK soybean plants were grown in growth chambers at 25 °C with 70% relative humidity and a 16/8 photoperiod.

### 2.2. ABA Treatment

The first trifoliate leaves of L29 and SMK plants (age ~16 days) were sprayed with ABA (100 µM) or mock (0.1% MeOH) 24 h before G7H-GFP or G5H-GFP inoculation. The plants received another ABA treatment 2 days post-infection (dpi), and samples were collected 5 dpi for analysis.

### 2.3. Virus Infection

The two unifoliate leaves from L29 and SMK plants were infected with 10 μg/leaf of pSMV-G7H-GFP or pSMV-G5H-GFP infectious clones [12,20]. A pool of systemically infected leaves from plants per virus strain were mixed and divided into 0.1 g quantities as a source of virus inoculum for each SMV strain. The 0.1 g quantities of infected tissues were ground to a powder in liquid nitrogen; mixed with 1 mL of phosphate buffer was vortexed with the ground 0.1 g infected tissues and then centrifuged for 10 min at 13,000 rpm. A 50 µL volume of the supernatant was rub-inoculated onto each leaf of the trifoliate on each plant, and samples were collected from three plants (total of 9 leaves) 5 dpi for further analyses.

### 2.4. Plant Sampling 

Experiments were carried out in three biological replicates, each replicate consisted of three plants, and one trifoliate leaf (which consists of 3 individual leaves) from each plant was infected with G7H-GFP or G5H-GFP. A pool of nine individual leaves was collected, cut and mixed, then 0.1 g of tissues were used for RNA extraction and protein synthesis. 

### 2.5. RNA Analysis

Total RNA was extracted using an RNA-extraction kit (Bio Cube, Suwon, South Korea) following the manufacturer’s instruction. A 1 μg quantity of total RNA was used for cDNA synthesis using the GoScript kit (Promega, Madison, WI, USA). Real-time quantitative reverse-transcription polymerase chain reaction (RT-qPCR) was carried out with SYBR-Green (Promega) to measure the relative expression of target genes using the ΔΔCT method. *Actin11* was used as an internal control, and the primers used in this study are listed in Table S1.

### 2.6. Protein Blot

Total protein was extracted from 0.1 g collected from a pool of inoculated leaves from three plants as described previously [11]. G7H-GFP and G5H-GFP were detected by protein blot using poly-clonal anti-GFP antibody (Sigma, St. Louis, MO, USA); Ponceau-S was used as the loading control.

### 2.7. Statistical Analysis

Error bars in the charts are means of standard deviation of three biological replicates. RT-qPCR was carried out in three biological replicates, and each biological replicate was repeated in technical replicates. In each panel, values were compared to that of the mock-treated, uninfected plants (the bar on the left) with one-sided Student’s *t*-tests; * and ** indicate a significant difference at *P* < 0.05 and <0.01, respectively.

### 2.8. Aniline Blue Staining

Callose was stained with aniline blue as described previously with slight modifications [21,22]. In brief, leaves were soaked in destaining lactophenol buffer (water: glycerol: phenol: lactic acid 1:2:2:1, with two volumes of 100% EtOH) for 24 h until the leaves became clear. Cleared leaves were then soaked in the staining buffer (0.01% aniline blue in 0.1 M phosphate buffer, pH 9.0) for 15 min. Leaves were washed twice with the destaining buffer for 10 min each time. Callose accumulation was observed with a microscope equipped with a DABI filter, with an excitation wavelength of 359 nanometers (nm), an emission wavelength of 461 nm, and an exposure time for 125 milliseconds.

### 2.9. Quantification of Callose

Three biological replicates (each consists of 12 leaf disks from 3 leaves) were used to observe callose and quantify its accumulation in L29 and SMK plants. The intensity of the blue fluorescence reflected from cell walls of soybean leaves was measured using Image J software as described previously [23] with slight modifications. Color images of blue fluorescence from leaf disks were converted to 8-bit. Integrated intensities of fluorescence (from the background and the cells) were divided by the covered areas to generate the average integrated densities (AID). AIDs of the background were subtracted from AIDs of the cells to obtain normalized readings of fluorescence intensities. Normalized readings from treated plants were divided by the normalized readings from untreated control plants and multiplied by 100 to obtain percentages.

### 2.10. Detection of H_2_O_2_ in Soybean Leaves

H_2_O_2_ was visually detected in the leaves as described previously [24,25]. In brief, soybean leaves were excised and directly immersed in DAB solution “3,3-diaminobenzidine” (0.5% DAB *w*/*v*, 1% HCl *v*/*v*, in PBS, pH 7.2) overnight. Leaves were then destained by soaking in 70% EtOH at 55 °C overnight. Cleared leaves with H2O2 stains were visualized using a Nikon D700 Camera. 

## 3. Results

### 3.1. ABA Induces Susceptibility to the Avirulent Strain G5H but Resistance to the Virulent Strain G7H in an Rsv3-Null Cultivar

**Figure 1:**
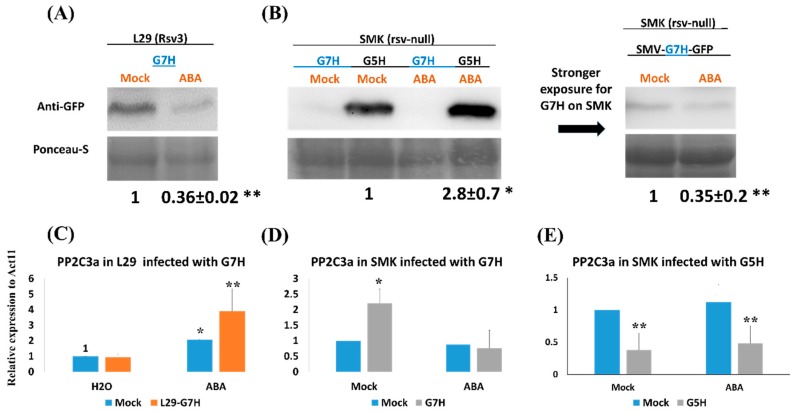
Effect of abscisic acid (ABA) treatment on the accumulation of soybean mosaic virus (SMV) strains and on *PP2C3a* expression in L29 and Somyungkong (SMK) soybean cultivars. Protein blots of the soybean mosaic virus (SMV) in response to exogenous application of ABA (100 μM) or Mock (0.1 % MeOH) in: (**A**) L29 cultivar (carries the *Rsv3* resistance gene) infected with the virulent strain G7H, or (**B**) SMK cultivar (rsv3-null) infected with G7H or the avirulent strain G5H (both strains express GFP). The upper panel shows the GFP level, and the lower panel shows Ponceau-S, which was used as a loading control. Relative expression levels of *PP2C3a* using RT-qPCR in response to G7H infection in L29 plants (**C**), G7H infection in SMK plants (**D**), and G5H plants (**E**). *Actin11* was used as the internal control. Plants were sampled at 5 dpi. For (**C**–**E**), values are means standard deviation (SD) of three biological replicates. In each panel, values were compared to that of the mock-treated, uninfected plants (the bar on the left) with one-sided Student’s *t*-tests; * and ** indicate a significant difference at *P* < 0.05 and <0.01, respectively.

To evaluate the efficiency of Rsv3-mediated defenses such as callose accumulation and the RNA-silencing pathway (which are partially regulated by ABA) against SMV strains in a line carrying dysfunctional *R* gene (which does not produce typical *R*-gene defense responses), we tested the susceptible soybean cultivar SMK against infection with the G5H and G7H strains of SMV [12]. In the G5H-resistant cultivar L29, which was used as a control, ABA decreased the accumulation of G7H by ~3-fold as expected (Figure 1A), but neither ABA nor G7H increased the expression of *Rsv3* (Figure S1). This indicated that G7H was not recognized by Rsv3 and that ABA probably acts downstream of Rsv3 recognition. We strongly suspect that the *Rsv3* gene in SMK cultivar has some deletions (or at least shares low similarities) compared with that of L29, because we were unable to clone this gene from SMK plants using primers that can amplify the full-length Rsv3 from L29 or even using other primers that can amplify various short regions (1 Kb) within the Rsv3 gene in L29 [26]. This implies that the *Rsv3* gene from SMK might be dysfunctional, or that these dissimilarities make rsv3 protein unable to recognize the CI region of the G5H and thus SMK cannot produce similar defense response to that observed in L29.

Interestingly, the SMK cultivar was more susceptible to the avirulent strain G5H than to the virulent strain G7H (Figure 1B). In addition, the effect of ABA on resistance against G5H was reversed in the SMK cultivar, i.e., accumulation of GFP was ~2.8-fold greater in ABA-treated than in the mock-treated SMK plants (Figure 1B). Still, and as noted earlier, ABA-induced resistance to G7H in SMK, i.e., ABA reduced G7H accumulation by ~3-fold relative to the mock treatment (Figure 1B, long-exposure western blot). We previously identified *PP2C3a* as a key gene for callose accumulation in the ABA pathway [8]. The expression of *PP2C3a* in L29 plants is rapidly induced in response to G5H infection but is not altered in response to G7H infection; when *PP2C3a* is co-expressed within the genome of the virulent strain G7H, callose accumulation is enhanced, and this restricts the spread of G7H at the point of infection [8]. As expected, ABA treatment of L29 plants increased the expression of *PP2C3a* by ~2-fold compared to mock-treated plants (Figure 1C). This increase approximately doubled when ABA-treated L29 plants were also infected with G7H (Figure 1C). In SMK plants, however, PP2C3a expression was not induced by ABA or the combination of ABA plus G7H infection, but was significantly induced by G7H infection in the absence of ABA treatment (Figure 1D). Interestingly, G5H infection decreased *PP2C3a* expression in SMK plants, and this expression profile was unaffected by ABA treatment (Figure 1E). These data suggest that ABA does not affect *PP2C3a* expression in the absence of the Rsv3 resistance responses and its related network.

### 3.2. Callose Accumulation, but Not Reactive Oxygen Species (ROS) Accumulation, Is Enhanced in Response to ABA in Rsv3-Cultivar L29

**Figure 2:**
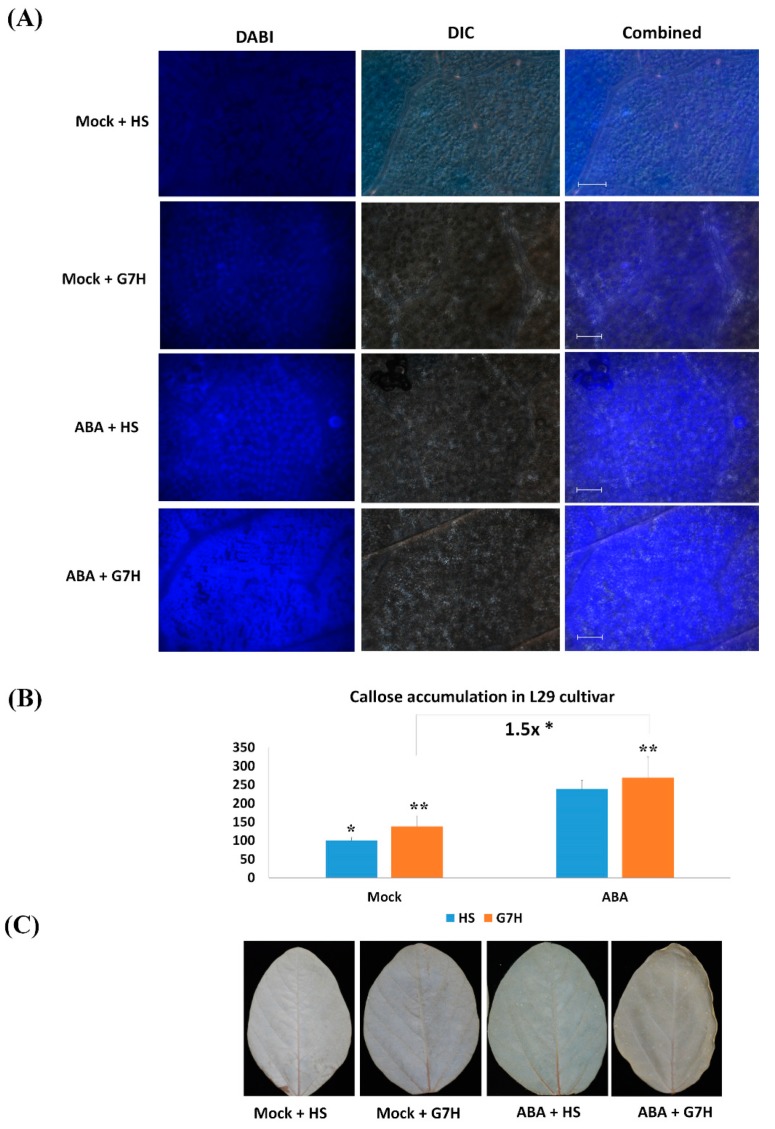
Levels of callose and reactive oxygen species (ROS) in L29 plants as affected by ABA treatment and G7H infection. L29 plants treated with Mock (0.1% MeOH) or with ABA (100 µM) were also treated with sap from healthy plants (HS) or sap from G7H-GFP-infected plants (G7H). Samples collected 5 dpi were subjected to aniline blue staining to reveal callose accumulation; scale bar = 20 µm (**A**). Callose fluorescence from aniline-blue-stained leaves was quantified using Image J software (**B**). ROS accumulation as indicated by DAB and as affected by ABA treatment, G7H-GFP infection, or both (**C**). The experiment was carried out in three independent replicates where values are means ± SD of three biological replicates, and statistical analysis was carried out as described in the legend of Figure 1, with additional comparison between Mock-G7H and ABA-G7H plants. * and ** indicate a significant difference at *P* < 0.05 and <0.01, respectively.

To confirm that ABA treatment induces callose, we next stained L29 leaves with aniline blue in order to assess the callose accumulation in response to ABA, and G7H infection. Significant accumulation of callose occurred in response to both ABA and G7H infection (1.3 and 2.4-fold, respectively), and the accumulation was even greater when both ABA and G7H were applied to L29 leaves (2.7 fold) (Figure 2A,B). The combined effect of ABA and G7H infection increased callose accumulation by ~1.5-fold compared with G7H alone (Figure 2B).

Previous reports indicated that ABA decreases the accumulation of hydrogen peroxide (H_2_O_2_) and other ROS in rice, and that this reduction negated the jasmonic acid (JA)-mediated resistance to rice black-streaked dwarf virus (RBSDV) [27]. Other reports indicated that increased ROS production in *Nicotiana tabacum* increases the acquisition of cucumber mosaic virus by aphids, and that ROS bursts are required for robust replication of red clover necrotic mosaic virus and brome mosaic virus [28,29]. We then used DAB staining to determine H_2_O_2_ levels and their possible roles in the responses of L29 plants to ABA and SMV. The staining revealed that neither ABA, G7H, nor their combination induced H_2_O_2_ in L29 plants (Figure 2C). This indicated that the ABA-mediated resistance in L29 plants does not rely on the induction of ROS.

### 3.3. Infection by the SMV Avirulent Strain G5H in Combination with ABA Treatment Reduces Callose Accumulation in SMK Plants

**Figure 3:**
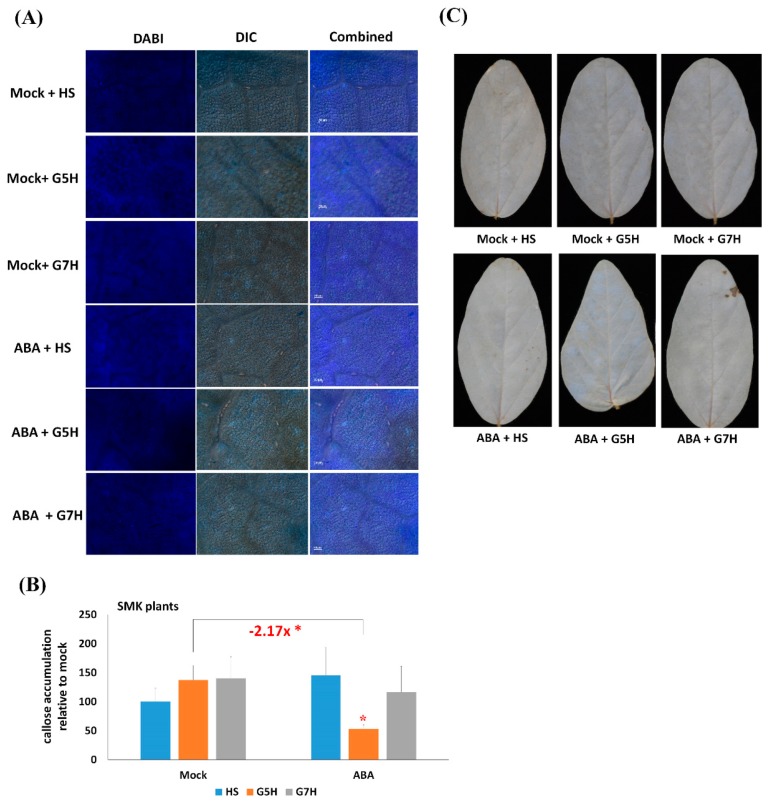
Levels of callose and ROS in SMK plants as affected by ABA treatment and G7H or G5H infection. SMK plants were treated with combinations of Mock (0.1% MeOH) or ABA (100 µM), and sap from healthy plants (HS), sap from G7H-GFP-infected plants (G7H), or sap from G5H-GFP-infected plants. Samples were collected 5 dpi and subjected to (**A**) aniline blue staining to detect callose accumulation levels, (**B**) quantification of callose fluorescence, and (**C**) DAB staining to reveal ROS accumulation. The experiment was carried out in three independent replicates, and values are means ± SD of three biological replicates. Statistical analysis was carried out as described in Figure legend 1, with additional comparison between Mock-G5H and ABA-G5H plants. Asterisks in red color indicate significant decrease at *P* < 0.05.

We next examined callose accumulation in SMK plants in response to infection with G7H or G5H, with or without ABA application. Infection with either strain did not significantly increase callose accumulation in SMK plants (Figure 3A,B). With ABA treatment, infection by G5H but not G7H significantly reduce callose accumulation (Figure 3A,B). Accumulation of an mRNA was downregulated 2.17-fold compared to the control (Figure 3B). It also appeared that SMV-G7H did not significantly reduce callose accumulation when it was applied with ABA, but this decrease was insignificant (Figure 3B). The accumulation of H_2_O_2_ in SMK plants resembled that of L29 plants in which neither SMV infection nor ABA treatment induced H_2_O_2_. These results suggest that G5H subverts the increase in defense caused by ABA treatment of the *rsv3*-null cultivar (SMK), perhaps because G5H alters some parts in the ABA-signaling pathway and thereby interferes with downstream defenses.

### 3.4. Several RNA-Silencing Genes in L29 Plants Are Induced in Response to ABA Treatment and G7H Infection

**Figure 4:**
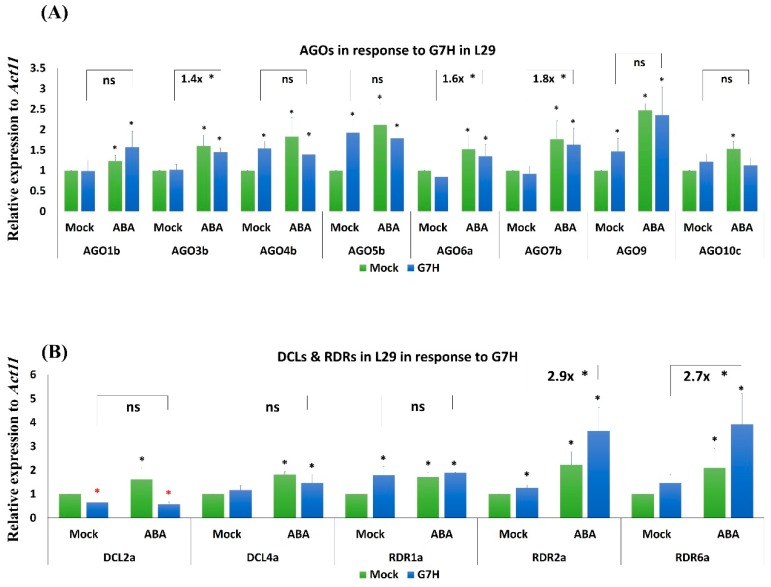
Expression levels of RNA-silencing genes in L29 plants infected with G7H. Expression levels of Argonaute (*AGO*) genes (**A**), and dicer like (*DCL*) and RNA polymerase (*RDR*) genes (**B**) in L29 plants in response to G7H infection, ABA treatment, or both. Plants were collected 5 dpi for RNA extraction and RT-qPCR. Values are means ± SD of three biological replicates. Statistical analysis was carried out for each gene using Student *t*-test; the means for plants treated with ABA alone, G7H alone, or the combination of ABA and G7H were individually compared with the mean for plants that were not treated with ABA or G7H: * and * indicate a significant increase or decrease, respectively, at *P* < 0.05. Additional analyses were carried for ABA-G7H in comparison with Mock-G7H where ns and * indicate non-significance and significance at *P* < 0.05, respectively.

In addition to callose induction, the induction of several *AGO* genes in the RNA-silencing pathway has been previously associated with ABA-mediated antiviral defense in *Arabidopsis* [15,17]. We therefore measured the effects of ABA treatment on the expression levels of eight *AGO* genes; in our previous study, these genes were selectively induced in response to G5H at an early stage of infection, and suggested that these *AGO* genes might contribute to the extreme resistance in L29 plants against G5H [11]. In addition to AGOs, we also tested other antiviral RNA-silencing genes from the DCL and RDR families. In response to G7H infection without ABA treatment, the expression levels of *GlymaAGO4a*, *GlymaAGO5b*, *GlymaAGO9*, *GlymaRDR1a*, and *GlymaRDR2a* were all significantly increased at 5 days post-infection (dpi) (Figure 4A). The expression of *GlymaAGO1b*, *GlymaAGO3b*, *GlymaAGO6a*, *GlymaAGO10c*, and *GlymaRDR6a* remained unchanged, and only *DCL2a* was down-regulated (Figure 4A,B). The effect of ABA alone but often caused significant increases in the expression of all genes (Figure 4A,B). Compared with G7H infection alone, the combination of ABA treatment and G7H infection significantly increased the expression of *GlymaAGO3b*, *GlymaAGO6a GlymaAGO7b*, *GlymaRDR2a*, and *RDR6a* by ~1.4-, 1.6-, 1.8-, 2.9-, and 2.7-fold, respectively (Figure 4A,B). The enhanced expression of these antiviral RNA-silencing genes suggests that ABA-mediated resistance is achieved through both callose accumulation and the induction of RNA-silencing pathways in the *Rsv3*-containing soybean cultivar L29.

### 3.5. Responses of the Antiviral RNA-Silencing Genes to ABA Are Weaker in the Rsv3-Null than in the Rsv3 Cultivar

**Figure 5:**
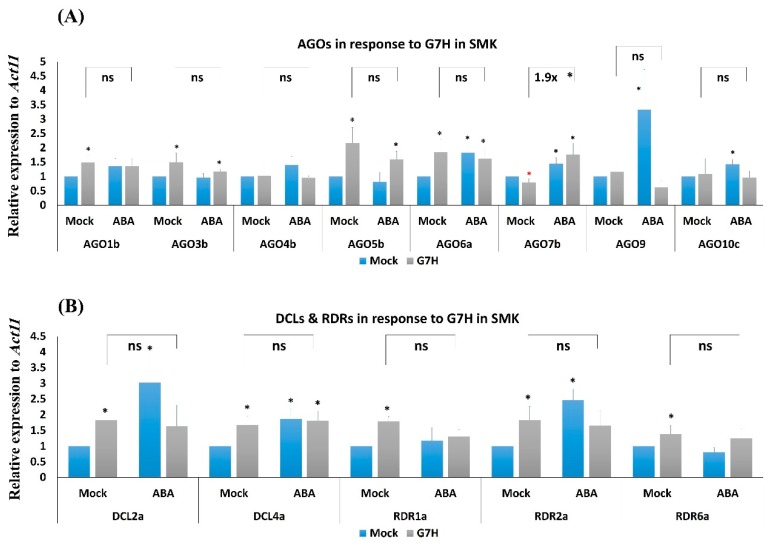
Expression levels of RNA-silencing genes in SMK plants infected with G7H. Expression levels of AGO genes (**A**), and DCL and RDR genes (**B**) in SMK soybean cultivar (*rsv3*-null) in response to G7H infection, ABA treatment, or both. Plants were collected 5 dpi for RNA extraction and RT-qPCR. Values are means ± SD of three biological replicates. Statistical analysis was carried out for each gene using Student *t*-test; the means for plants treated with ABA alone, G7H alone, or the combination of ABA and G7H were individually compared with the mean for plants that were not treated with ABA or G7H: * and * indicate a significant increase or decrease, respectively, at *P* < 0.05. Additional analyses were carried for ABA-G5H in comparison with Mock-G7H where ns and * indicate non-significance and significance at *P* < 0.05, respectively.

Although ABA treatment increased the expression of all of the tested RNA-silencing genes in L29 plants (Figure 4), ABA treatment significantly increased the expression of only *AGO6a*, *AGO7b*, *AGO10c*, *DCL2a*, and *RDR2a* in SMK plants, and the increase for these genes was generally weaker than in L29 plants (Figure 5). Among all tested *AGO* genes in SMK plants, G7H increased the expression of only *AGO3b*, *AGO5b*, and *AGO6a* and reduced the expression of only *AGO7b* (Figure 5A). G7H infection, however, increased the expression of all *DCL* and *RDR* genes in SMK plants (Figure 5B). The combined effect of ABA treatment and G7H infection led to a significant increase in the expression of one gene, *AGO7b*, in SMK plants (Figure 5A). These results suggest that the absence of the *Rsv3* resistance response affects the expression of several RNA-silencing genes, and that this absence may also influence other downstream genes in the *Rsv3*-related defense responses.

### 3.6. Infection with G5H Reverses the Effect of ABA on the Expression of Several RNA-Silencing Genes in SMK Plants

**Figure 6:**
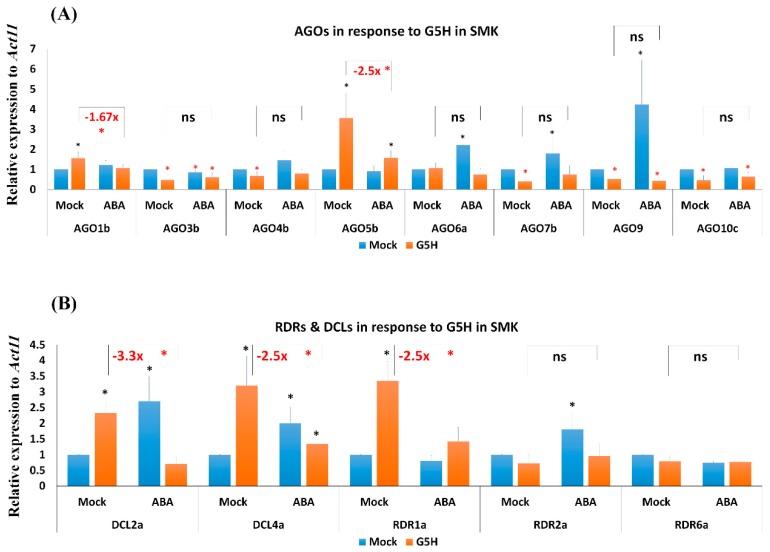
Expression levels of RNA-silencing genes in SMK plants infected with G5H. Expression levels of *AGO* genes (**A**), and *DCL* and *RDR* genes (**B**) in SMK soybean cultivar (*rsv3*-null) in response to G5H infection, ABA treatment, or both. Plants were collected 5 dpi for RNA extraction and RT-qPCR. Data are means ± standard deviation from three biological replicates. Statistical analysis was carried out for each gene using Student *t*-test; the means for plants treated with ABA alone, G5H alone, or the combination of ABA and G5H were individually compared with the mean for plants that were not treated with ABA or G5H: * and * indicate a significant increase or decrease, respectively, at *P* < 0.05. Additional analyses were carried for ABA-G5H in comparison with Mock-G5H where ns and * indicate non-significance and significance at *P* < 0.05, respectively.

SMK plants responded to infection by G5H with increased expression of *AGO1b*, *AGO5b*, *DCL2a*, *DCL4a*, and *RDR1a* (Figure 6A,B). Interestingly, G5H infection significantly reduced the expression of *AGO3b*, *AGO4b*, *AGO7b*, *AGO9*, and *AGO10c* (Figure 6A,B). The combination of ABA treatment and G5H infection significantly downregulated the expression of *AGO1b*, *AGO5b*, *DCL2a*, *DCL4a*, and *RDR1a* with ~1.67-, 2.5-, 3.3-, 2.5-, and 2.5-fold, respectively (Figure 6). These results suggest that the avirulent strain G5H reverses the effect of ABA on the expression of several RNA-silencing genes in the *rsv3*-null plants, and that this reduction may contribute to the increased susceptibility to G5H following ABA treatment.

## 4. Discussion

In several dicots, ABA contributes to defense against viruses [13,14,17]. The positive effect of ABA on virus resistance has been attributed to enhanced callose deposition at plasmodesmata and enhanced expression of several genes in the antiviral RNA-silencing pathways [6,15,16,17]. Most reports concerning these ABA effects involve compatible plant–virus interactions. Only one report assessed the effect of ABA on an incompatible interaction in plants with specific *R*-resistance genes, and that report concerned soybean cultivar L29, which carries the *Rsv3* ER gene [3,8]. Once Rsv3 recognizes the CI in the G5H strain of SMV, a cascade of defense arrays is triggered including autophagy, the RNA-silencing pathway, and the ABA-signaling pathway, in addition to the suppression of several genes in the jasmonic acid pathway and in the WRKY gene family [11]. In the compatible interaction between L29 plants and G7H, these responses were absent or weak, and SA and its related genes were induced but only several days after infection [8]. This is a typical sign of the stress-induced incompatible plant–virus interactions [30,31,32]. We found that the induction of all of the assessed RNA-silencing genes was increased in *Rsv3* plants when they were treated with ABA (Figure 3). In SMK plants (rsv3-null), in contrast, the effect of ABA was limited to *AGO6a*, *AGO7b*, *DCLs*, and *RDR1* (Figure 4). Previous reports indicated that all DCLs in *Arabidopsis* are affected by ABA because their mutants are hypersensitive to ABA [33] and because they are commonly expressed following ABA treatment [16]. These findings suggest a role for Rsv3 in enhancing ABA regulation of the expression of *AGO* genes in particular, and of the RNA-silencing pathway in general. 

Interestingly, ABA failed to induce *PP2C3a* in SMK plants (Figure 1D) but increased the expression in L29 by 2-fold, and that effect becomes even stronger with ABA and G7H infection on L29 with ~4 fold (Figure 1C). These findings are consistent with the inference that the defensive effects ABA are more prominent with the *Rsv3* gene (and its related network) in the L29 genetic background than in rsv3-null plants. Notably, callose accumulation was decreased in SMK plants infected with G5H although (Figure 1E). The downregulation of *PP2C3a* might be a result of G5H disruption of the ABA signaling pathway, which would explain why exogenous ABA had no effect on *PP2C3a* expression in SMK plants infected with G5H. Unlike G5H, G7H infection did induce *PP2C3a* expression in the absence of ABA treatment of SMK plants (Figure 1D). This suggests that G7H infection regulates *PP2C3a* in both an ABA-dependent and an ABA-independent manner. These observations are consistent with callose accumulation in SMK plants that were infected with G7H and treated with ABA, where callose accumulation was similar to that found in healthy tissue. ABA also mediates callose deposition by regulating β,1-3 glucanases, which degrade callose [34]. When combined with ABA treatment, G5H infection might reduce callose accumulation by interfering with callose synthase genes or by enhancing the activity of β,1-3 glucanases (Figure 3). 

The ability of G5H to invert ABA-mediated defenses was evident for the expression of the RNA silencing genes where several AGOs and DCLs were significantly downregulated in response to SMV-G5H infection in ABA-treated SMK plants (Figure 6). This suggests that, in the absence of functional *Rsv3*, G5H can alter or interfere the expression, function, or activity of regulators in the ABA-signaling pathway, such that the effect of ABA is the opposite in G5H-infected plants than in G7H-infected plants. Interfering the ABA signaling pathway might be a way for some viral strains to increase the susceptibility of the host. For example, the RNA silencing suppressor 2b of the cucumber mosaic virus interferes with the ABA pathway in *Arabidopsis* where *ABA*-related genes are downregulated in 2b-transgenic *Arabidopsis* but with high tolerance to drought through increased stomatal conductance compared with nontransgenic plants [35]. In addition, suppression of the ABA pathway increases rice susceptibility to infection with RBSDV [27]. Several factors affect the NBS-LRR network, and these factors act on levels ranging from the transcriptional to the posttranslational [8,36]. EXA1, for example, is a negative regulator of NBS-LRR accumulation [37]. Similarly, RACK1 is a negative regulator in the ABA-signaling pathway in *Arabidopsis*, and its mutant increases the expression of ABA-responsive genes [38]. ROP11 is also a negative regulator of ABA responses, and ABA-mediated responses are downregulated when ROP11 is constitutively expressed [39]. Other negative regulators of the ABA signaling pathway include members of the PP2C clade, such as ABI1, ABI2, and PP2C3a [40]. ABA perception by the receptor PYR/PYL/RCAR requires specific PP2C proteins (ABI1 and ABI2) to form one large complex. This perception releases SnRK2 from PP2Cs, and SnRK2 then becomes available to phosphorylate ABA-related transcription factors that transcribe ABA-responsive genes [41]. It is likely that G5H has the ability to interfere with such key regulators of the ABA-signaling pathway, and that this interference reverses the effect of ABA on its responsive genes. Besides, components of the NBS-LRR network; the sensor, the helpers, could also differ between L29 and SMK cultivars. We were unable to clone *Rsv3* genes from SMK plants using several pairs of primers designed based on the *Rsv3*-template from L29. This implies that it is highly possible that the SMK cultivar lacks a functional *Rsv3* gene due to dissimilarities or some deletions within the coding region. How G5H is able to interfere with ABA signaling pathways and the ABA-regulated resistance mechanisms requires further investigation.

Xie et al. [27] reported that ABA reduced ROS levels and interfered with JA-mediated defense against RBSDV [27]. Our findings appear to rule out any possible effect of ABA on ROS because ROS levels were not altered in response to ABA treatment even when combined with infection by G7H or G5H. 

In summary, the current results suggest that the resistance of the L29 plants depends on the *Rsv3* gene and its genetic network, and that the absence (or dysfunction) of *Rsv3* in SMK plants, and the apparent ability of G5H to interfere with the downstream resistance network may explain the increased susceptibility of ABA-treated SMK plants to G5H infection [36]. Further research on how G5H infection alters susceptible cultivars and how the defense networks are changed in the absence of a sensor *R*-gene will increase our understanding of how defense networks operate against virulent vs. avirulent strains of SMV and other viruses.

## Supplementary Materials

The following are available online at https://www.mdpi.com/1999-4915/11/9/879/s1, Figure S1. Expression levels of *Rsv3* gene in response to G7H infection, ABA treatment, or both. The first trifoliate leaf was sprayed with ABA one day before G7H infection, and received another treatment after three days. Leaves were collected at 5 dpi for expression analysis.

## References

[1] Liu J.Z., Fang Y., Pang H. (2016). The Current Status of the Soybean-Soybean Mosaic Virus (SMV) Pathosystem. Front. Microbiol..

[2] Hajimorad M.R., Domier L.L., Tolin S.A., Whitham S.A., Saghai Maroof M.A. (2018). Soybean mosaic virus: A successful potyvirus with a wide distribution but restricted natural host range. Mol. Plant Pathol..

[3] Tran P.T., Widyasari K., Seo J.K., Kim K.H. (2018). Isolation and validation of a candidate Rsv3 gene from a soybean genotype that confers strain-Specific resistance to soybean mosaic virus. Virology.

[4] Klepadlo M., Chen P.Y., Shi A.N., Mason R.E., Korth K.L., Srivastava V., Wu C.J. (2017). Two Tightly Linked Genes for Soybean Mosaic Virus Resistance in Soybean. Crop Sci..

[5] Gunduz I., Buss G.R., Ma G., Chen P., Tolin S.A. (2001). Genetic analysis of resistance to soybean mosaic virus in OX670 and harosoy soybean. Crop Sci..

[6] Reagan B.C., Ganusova E.E., Fernandez J.C., McCray T.N., Burch-Smith T.M. (2018). RNA on the move: The plasmodesmata perspective. Plant Sci..

[7] Ma F.F., Wu M., Liu Y.N., Feng X.Y., Wu X.Z., Chen J.Q., Wang B. (2018). Molecular characterization of NBS-LRR genes in the soybean Rsv3 locus reveals several divergent alleles that likely confer resistance to the soybean mosaic virus. Theor. Appl. Genet..

[8] Seo J.K., Kwon S.J., Cho W.K., Choi H.S., Kim K.H. (2014). Type 2C Protein Phosphatase Is a Key Regulator of Antiviral Extreme Resistance Limiting Virus Spread. Sci. Rep..

[9] Zhang C.Q., Hajimorad M.R., Eggenberger A.L., Tsang S., Whitham S.A., Hill J.H. (2009). Cytoplasmic inclusion cistron of Soybean mosaic virus serves as a virulence determinant on Rsv3-Genotype soybean and a symptom determinant. Virology.

[10] Seo J.K., Lee S.H., Kim K.H. (2009). Strain-Specific Cylindrical Inclusion Protein of Soybean mosaic virus Elicits Extreme Resistance and a Lethal Systemic Hypersensitive Response in Two Resistant Soybean Cultivars. Mol. Plant Microbe. Interact..

[11] Alazem M., Tseng K.C., Chang W.C., Seo J.K., Kim K.H. (2018). Elements Involved in the Rsv3-Mediated Extreme Resistance against an Avirulent Strain of Soybean Mosaic Virus. Viruses.

[12] Seo J.K., Lee H.G., Choi H.S., Lee S.H., Kim K.H. (2009). Infectious in vivo Transcripts from a Full-Length Clone of Soybean mosaic virus Strain G5H. Plant Pathol. J..

[13] Alazem M., Lin K.Y., Lin N.S. (2014). The Abscisic Acid Pathway Has Multifaceted Effects on the Accumulation of Bamboo mosaic virus. Mol. Plant Microbe Interact..

[14] Alazem M., Lin N.S. (2015). Roles of plant hormones in the regulation of host-Virus interactions. Mol. Plant Pathol..

[15] Alazem M., He M.H., Moffett P., Lin N.S. (2017). Abscisic Acid Induces Resistance against Bamboo Mosaic Virus through Argonaute2 and 3. Plant Physiol..

[16] Alazem M., Kim K.H., Lin N.S. (2019). Effects of Abscisic Acid and Salicylic Acid on Gene Expression in the Antiviral RNA Silencing Pathway in Arabidopsis. Int. J. Mol. Sci..

[17] Alazem M., Lin N.S. (2017). Antiviral Roles of Abscisic Acid in Plants. Front. Plant Sci..

[18] Adachi H., Derevnina L., Kamoun S. (2019). NLR singletons, pairs, and networks: Evolution, assembly, and regulation of the intracellular immunoreceptor circuitry of plants. Curr. Opin. Plant Biol..

[19] Wu C.H., Derevnina L., Kamoun S. (2018). Receptor networks underpin plant immunity. Science.

[20] Seo J.K., Lee H.G., Kim K.H. (2009). Systemic gene delivery into soybean by simple rub-Inoculation with plasmid DNA of a Soybean mosaic virus-Based vector. Arch. Virol..

[21] Schenk S.T., Hernandez-Reyes C., Samans B., Stein E., Neumann C., Schikora M., Reichelt M., Mithofer A., Becker A., Kogel K.H. (2014). N-Acyl-Homoserine Lactone Primes Plants for Cell Wall Reinforcement and Induces Resistance to Bacterial Pathogens via the Salicylic Acid/Oxylipin Pathway. Plant Cell.

[22] Li W.L., Zhao Y.S., Liu C.J., Yao G.B., Wu S.S., Hou C.Y., Zhang M.C., Wang D.M. (2012). Callose deposition at plasmodesmata is a critical factor in restricting the cell-to-Cell movement of Soybean mosaic virus. Plant Cell Rep..

[23] Zavaliev R., Epel B.L., Heinlein M. (2015). Imaging Callose at Plasmodesmata Using Aniline Blue: Quantitative Confocal Microscopy. Plasmodesmata: Methods and Protocols.

[24] Orozco-Cardenas M., Ryan C.A. (1999). Hydrogen peroxide is generated systemically in plant leaves by wounding and systemin via the octadecanoid pathway. Proc. Natl. Acad. Sci. USA.

[25] Chen X.R., Wang X.L., Zhang Z.G., Wang Y.C., Zheng X.B. (2008). Differences in the induction of the oxidative burst in compatible and incompatible interactions of soybean and Phytophthora sojae. Physiol. Mol. Plant Pathol..

[26] Alazem M., Kim K.H. (2019). Rsv3 Amplification from L29 and Somyungkong Soybean Cultivars.

[27] Xie K., Li L., Zhang H., Wang R., Tan X., He Y., Hong G., Li J., Ming F., Yao X. (2018). Abscisic acid negatively modulates plant defence against rice black-Streaked dwarf virus infection by suppressing the jasmonate pathway and regulating reactive oxygen species levels in rice. Plant Cell Environ..

[28] Guo H.J., Gu L.Y., Liu F.Q., Chen F.J., Ge F., Sun Y.C. (2019). Aphid-Borne Viral Spread Is Enhanced by Virus-Induced Accumulation of Plant Reactive Oxygen Species. Plant Physiol..

[29] Hyodo K., Hashimoto K., Kuchitsu K., Suzuki N., Okuno T. (2017). Harnessing host ROS-Generating machinery for the robust genome replication of a plant RNA virus. Proc. Natl. Acad. Sci. USA.

[30] Zhou T., Murphy A.M., Lewsey M.G., Westwood J.H., Zhang H.M., Gonzalez I., Canto T., Carr J.P. (2014). Domains of the cucumber mosaic virus 2b silencing suppressor protein affecting inhibition of salicylic acid-Induced resistance and priming of salicylic acid accumulation during infection. J. Gen. Virol..

[31] Baebler S., Stare K., Kovac M., Blejec A., Prezelj N., Stare T., Kogovsek P., Pompe-Novak M., Rosahl S., Ravnikar M. (2011). Dynamics of responses in compatible potato-Potato virus Y interaction are modulated by salicylic acid. PLoS ONE.

[32] Huang Z., Yeakley J.M., Garcia E.W., Holdridge J.D., Fan J.B., Whitham S.A. (2005). Salicylic acid-Dependent expression of host genes in compatible Arabidopsis-Virus interactions. Plant Physiol..

[33] Zhang J.F., Yuan L.J., Shao Y., Du W., Yan D.W., Lu Y.T. (2008). The disturbance of small RNA pathways enhanced abscisic acid response and multiple stress responses in Arabidopsis. Plant Cell Environ..

[34] Oide S., Bejai S., Staal J., Guan N., Kaliff M., Dixelius C. (2013). A novel role of PR2 in abscisic acid (ABA) mediated, pathogen-Induced callose deposition in Arabidopsis thaliana. New Phytol..

[35] Westwood J.H., McCann L., Naish M., Dixon H., Murphy A.M., Stancombe M.A., Bennett M.H., Powell G., Webb A.A., Carr J.P. (2013). A viral RNA silencing suppressor interferes with abscisic acid-Mediated signalling and induces drought tolerance in Arabidopsis thaliana. Mol. Plant Pathol..

[36] Gallois J.L., Moury B., German-Retana S. (2018). Role of the Genetic Background in Resistance to Plant Viruses. Int. J. Mol. Sci..

[37] Wu Z.S., Huang S.A., Zhang X.B., Wu D., Xia S.T., Li X. (2017). Regulation of plant immune receptor accumulation through translational repression by a glycine-tyrosine-Phenylalanine (GYF) domain protein. Elife.

[38] Guo J.J., Wang J.B., Xi L., Huang W.D., Liang J.S., Chen J.G. (2009). RACK1 is a negative regulator of ABA responses in Arabidopsis. J. Exp. Bot..

[39] Li Z.X., Kang J., Sui N., Liu D. (2012). ROP11 GTPase is a Negative Regulator of Multiple ABA Responses in Arabidopsis. J. Integr. Plant Biol..

[40] Bhaskara G.B., Wong M.M., Verslues P.E. (2019). The flip side of phospho-Signalling: Regulation of protein dephosphorylation and the protein phosphatase 2Cs. Plant Cell Environ..

[41] Gonzalez-Guzman M., Pizzio G.A., Antoni R., Vera-Sirera F., Merilo E., Bassel G.W., Fernandez M.A., Holdsworth M.J., Perez-Amador M.A., Kollist H. (2012). Arabidopsis PYR/PYL/RCAR receptors play a major role in quantitative regulation of stomatal aperture and transcriptional response to abscisic acid. Plant Cell.

