# Supplementary material for: An Avirulent Strain of Soybean Mosaic Virus Reverses the Defensive Effect of Abscisic Acid in a Susceptible Soybean Cultivar
Source: Viruses. 2019 Sep 19;11(9):879. doi: 10.3390/v11090879 (PMC6783863; doi:10.3390/v11090879)
Supplement: Supplementary file 1 [file viruses-11-00879-s001.pdf]

Fig. S1. Expression levels of *Rsv3* gene in response to G7H infection, ABA treatment, or both. The first trifoliate leaf was sprayed with ABA one day before G7H infection, and received another treatment after three days. Leaves were collected at 5 dpi for expression analysis.

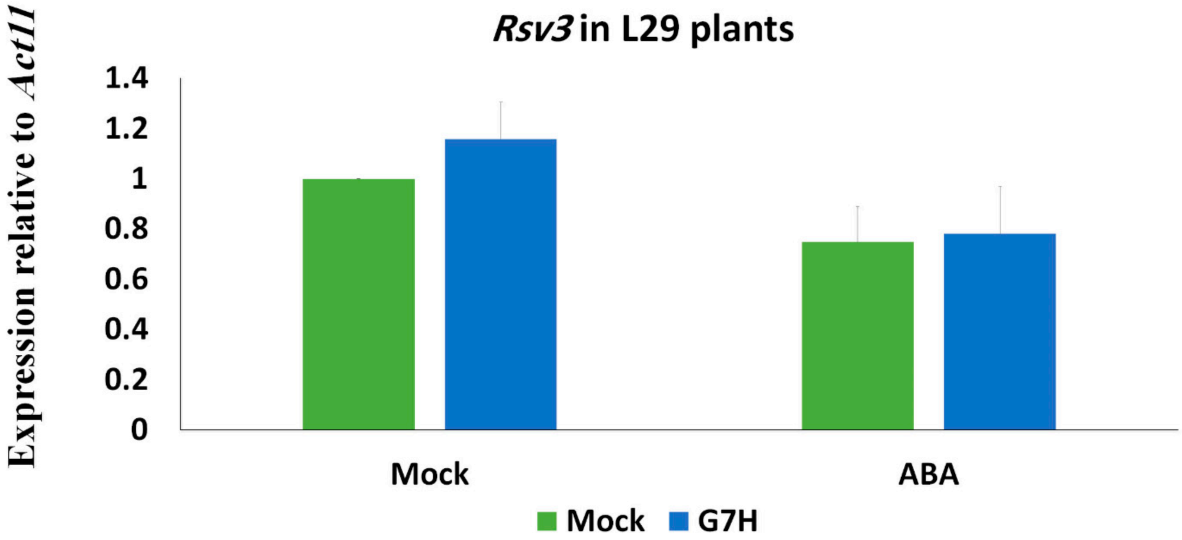

Table S1. The primers used in this study.

Gene Name	Fw (5'→3')	Rv (5'→3')
AGO1b	CTCACCTTGCTGCATTCCGT	TGCAACAGCACCACTTGTCA
AGO3b	TGAAGCAAGGATCAGGATGCA	TCCAGGTCAGCATGCAATGT
AGO5b	AGGAACTAGTCGACCAACACA	TCGAGTACACCTTGCATACGT
AGO6a	TTGGTTTCTCGGCAGATGGC	GCAGCATGGTGAGCATAGCA
AGO7b	ATTTCTTTGGTGCCTCCTGC	GGTGCTGTCTTTGGAGGAGC
AGO9	TGGGTTCGCGGTATTGGTTG	TCTTCCTCCTCCTGCAACCC
AGO10c	GCGCGGTTCTATATGGAGCC	TCTTGTTGCCTTGGAGCCAC
DCL2a	GGCGGTGCTCATAAGGACAC	ACCCTTGTGCACAGTACACA
DCL4a	GAGGGACCAGACCACCTGAA	CCATAATGCACCCTCTGCCG
RDR1a	TCCAAGTTACTGGGGTTGCT	ACGCAACCCACTGAAACTGT
RDR2a	GGTAACGTGCAAAACCGTGC	ACGCGGTTGGCAACTAGTTT
RDR6a	CAGTTGATTACCTAGCTCGC	GAGCAAGCTCTCAATGGAAT
PP2C3a	TGAAGTTGCGTGCCAAGTTG	TCGGTAGCACGGTTTTGATG
Actin	ATCTTGACTGAGCGTGGTTATTCC	GCTGGTCCTGGCTGTCTCC
